# Effects of *Hericium erinaceus* Mycelium Extracts on the Functional Activity of Purinoceptors and Neuropathic Pain in Mice with L5 Spinal Nerve Ligation

**DOI:** 10.1155/2020/2890194

**Published:** 2020-05-13

**Authors:** Pao-Pao Yang, Sheau-Huei Chueh, Hua-Lun Shie, Chin-Chu Chen, Li-Ya Lee, Wan-Ping Chen, Yu-Wen Chen, Li-yen Shiu, Pei-Shan Liu

**Affiliations:** ^1^Department of Biochemistry, National Defense Medical Center, Taipei, Taiwan; ^2^Department of Microbiology, Soochow University, Taipei, Taiwan; ^3^Biotechnology Center, Grape King Bio Ltd., Taoyuan 320, Taiwan; ^4^Cell Therapy and Research Center, Department of Medical Research, E-Da Cancer Hospital, Kaohsiung, Taiwan

## Abstract

Neuropathic pain is a serious clinical problem that is difficult to treat. Purinoceptors (P2Rs) transduce pain perception from the peripheral to the central nervous system and play an important role in the transmission of neuropathic pain signals. We previously found that the crude extracts of *Hericium erinaceus* mycelium (HE-CE) inhibited P2R-mediated signaling in cells and reduced heat-induced pain in mice. The present study explored the effects of HE-CE on neuropathic pain. We used adenosine triphosphate (ATP) as a P2R agonist to generate Ca^2+^ signaling and neuronal damage in a cell line. We also established a neuropathic mouse model of L5 spinal nerve ligation (L5-SNL) to examine neuropathic pain and neuroinflammation. Neuropathic pain was recorded using the von Frey test. Neuroinflammation was evaluated based on immunohistofluorescence observation of glial fibrillary acidic protein (GFAP) levels in astrocytes, ionized calcium-binding adaptor molecule1 (iba1) levels in microglia, and IL-6 levels in plasma. The results show that HE-CE and erinacine-S, but not erinacine-A, totally counteracted Ca^2+^ signaling and cytotoxic effects upon P2R stimulation by ATP in human osteosarcoma HOS cells and human neuroblastoma SH-SY5Y cells, respectively. SNL induced a decrease in the withdrawal pressure of the ipsilateral hind paw, indicating neuropathic pain. It also raised the GFAP level in astrocytes, the iba1 level in microglia, and the IL-6 level in plasma, indicating neuroinflammation. HE-CE significantly counteracted the SNL-induced decrease in withdrawal pressure, illustrating that it could relieve neuropathic pain. It also reduced SNL-induced increases in astrocyte GFAP levels, microglial iba1 levels, and plasma IL-6 levels, suggesting that HE-CE reduces neuroinflammation. Erinacine-S relieved neuropathic pain better than HE-CE. The present study demonstrated that HE inhibits P2R and, thus, that it can relieve neuropathic pain and neuroinflammation.

## 1. Introduction

Pain is a sensation triggered in the nervous system in response to the stimulation of the purinoceptor (P2R) by adenosine triphosphate (ATP). When cells are damaged or stressed, ATP is released from either the sensory neurons themselves [1] or from the adjacent peripheral tissue [2]. Extracellular ATP activates P2Rs in the nociceptive pathways, both at their peripheral and central terminals in the spinal cord [2–6]. These P2Rs, which are categorized as including ionotropic P2X receptors (P2XRs) and metabotropic P2Y receptors (P2YRs), then generate and modulate various forms of pain [3,6,7]. Abundant evidence suggests that P2Rs are important in the transmission of neuropathic pain [7,8], which is the most debilitating of all clinical pain syndromes. Such pain results from nerve injury due to surgery, diabetes, cancer, or infection in the central or peripheral nervous system [9]. Neuropathic pain is generally resistant to currently available treatments and can be very difficult to alleviate, with only 40% of patients showing partial relief [9]. Thus, safe and effective treatments for relieving neuropathic pain are urgently needed. In this regard, P2R antagonists protect against neuropathic pain [10] and may therefore guide the search for analgesic medicine in patients with neuropathic pain.

Spinal nerve ligation (SNL) in rodent was first described in 1992 [11] and used as a neuropathic pain model [12–15]. SNL surgery induces severe mechanical allodynia, as evidenced by the decreased hind paw withdrawal threshold during von Frey hair stimulation. The surgery also leads to immediate postoperative pain and results in prolonged mechanical allodynia. As such, it may be a good model for studying neuropathic pain. Interleukin-6 (IL-6) is an inflammatory cytokine whose levels rise during nerve damage. SNL surgery induces spinal hypertrophy with increased expression of glial fibrillary acidic protein (GFAP) in astrocytes and of the ionized calcium-binding adaptor molecule 1 (iba1) in microglia [14], indicating activation of these cells. Activated astrocytes and microglia also play a role in the initiation and maintenance of neuropathic pain after SNL surgery [16,17]. In the present study, we explored mechanical allodynia, activation of spinal astrocytes and microglia, and plasma IL-6 levels following SNL surgery, seeking to better understand the progression of neuropathic pain.

The use of natural compounds that antagonize nociceptive transmission by P2Rs has been proposed as a strategy for safe and effective relief of neuropathic pain [18]. In addition to neurotransmission, strong activation of P2R can cause Ca^2+^ overload and consequent cell death [19,20]. Antagonists of P2R can rescue neurons from P2R-mediated neurotransmission and neurotoxicity [20]. We previously found that crude extracts of *Hericium erinaceus* (HE-CE) suppressed P2R-related Ca^2+^ signaling and thermal pain [21]. Erinacine-A (E-A), a cyanthin diterpenoid, and erinacine-S (E-S), a sesterterpene, are two major components of HE-CE [22–25]. The present study investigated the inhibitory effects of HE-CE, E-A, and E-S on P2R signaling, as well as whether these substances can relieve SNL-induced neuropathic pain and neuroinflammation.

## 2. Materials and Methods

### 2.1. Materials

*Hericium erinaceus* (BCRC 35669) was sourced from the Bioresources Collection and Research and Development Institute (Hsinchu, Taiwan). ATP, HEPES, NaHCO3, digitonin, ethylene glycol tetra-acetic acid (EGTA), and fura-2/AM were purchased from Sigma-Aldrich Co. (St. Louis, MO, USA). Dulbecco's modified Eagle's medium and penicillin-streptomycin were purchased from Gibco BRL (Gaithersburg, MD, USA). HE-CE, E-A, and E-S were obtained from Grape King Bio Ltd.

### 2.2. High-Performance Liquid Chromatography (HPLC) Analysis

We analyzed the chemical composition of HE-CE using HPLC (Thermo Scientific Vanquish Horizon UHPLC System), while E-A and E-S were separated using a COSMOSIL 5C18-AR-II column (250 × 4.6 mm; particle size 5 *μ*m, Nacalai USA, Inc.) [22–25].

### 2.3. Preparation of HE-CE, E-A, and E-S

HE-CE was prepared following the method described by Hu et al. [22–24]. In brief, 95% ethanol was added to the *Hericium erinaceus* mycelium powder and the preparation was ultrasonicated for 2 hours. The resulting solution was filtered and concentrated under a vacuum to obtain a brown extract, which was then partitioned through a 1 : 1 solution of water/ethyl acetate. The ethyl acetate layer was analyzed using silica gel column chromatography (70–230 mesh; 70 × 10 cm), and a 3 : 2 solution of *n*-hexane/ethyl acetate was used to perform gradient separation. E-S was obtained using rechromatography on a Sephadex LH-20 column and a silica column [24]. Finally, fractions collected using *n*-hexane/ethyl acetate (1 : 2) were sequentially eluted using methanol, 70% methanol through a Sephadex LH-20 column, and then 60% methanol using an RP-18 column to obtain E-A [25].

### 2.4. Cell Culture

Human neuroblastoma SH-SY5Y cells, obtained from ATCC (CRL-2266), were cultured as described previously [26], as were human osteosarcoma (HOS) cells, obtained from the Bioresource Collection and Research Center (Hsinchu, Taiwan) [27].

### 2.5. Cytosolic Free Ca^2+^ Concentration ([Ca^2+^]_c_) Measurement

We measured [Ca^2+^]_c_ using fura-2 Ca^2+^ fluorescent dye, following our previous methods [27]. In brief, cells were loaded with 10 *μ*M fura-2 at 37°C for 40 minutes; they were then washed twice in loading buffer containing 150 mM NaCl, 5 mM KCl, 5 mM glucose, 1 mM MgCl_2_, 2.2 mM CaCl_2_, and 10 mM HEPES 10 (pH 7.4). Fluorescent measurements were performed using a dual-excitation fluorimeter (SPEX; CM systems) at 340 and 380 nm excitation and 505 nm emission. The[Ca^2+^]_c_ was calculated based on the fluorescence ratio between 340 nm and 380 nm excitation. *R*max was achieved by adding 0.01% digitonin to the cuvette at the end of experiments; excess EGTA was subsequently added to obtain *R*min. A *K*d of 224 nM Ca^2+^ was used for fura-2. Each data point represents the results of five individual experiments for each protocol, using batches of cells, and each experiment was carried out at least in duplicate.

### 2.6. MTT Assay

We carried out the MTT assay following our previous method [27]. In brief, we cultured the cells in a 96-well plate with a density of 1 × 10^4^ cells/well. After the treatments and washings, we added 0.4 mg/mL 3-(4,5-dimethylthiazol-2-yl)-2,5-diphenyltetrazolium bromide (MTT) into the 96-well plate for 4 hours. Next, we added DMSO to dissolve the precipitate. The optical density of each well was read at 595 nm, with a reference at 650 nm, on an ELISA reader (Molecular Devices VersaMax, CA, USA). We used five batches of cells for each experiment, and each was carried out in triplicate.

### 2.7. Ethical Statement

All experimental procedures were approved by the Institutional Animal Care and Use Committee at the Animal Center of the National Defense Medical Center, Taiwan, which is accredited by AAALAC International.

### 2.8. Animals

Male C57BL/6 NARL mice weighing 20–25 grams and aged 6–8 weeks during the experiments were obtained from the National Laboratory Animal Center of Taiwan. The mice were kept in the animal rooms at the Animal Center of Taiwan's National Defense Medical Center. The institutional and international standards were followed for the care of all animals (Principles of Laboratory Animal Care, NIH), and the protocol was approved by the Institutional Animal Care and Use Committee (IACUC) of the National Defense Medical Center, Taiwan. All studies involving animals are reported in accordance with the ARRIVE guidelines [28].

### 2.9. SNL Surgery

We carried out L5-SNL surgery according to a previously described method for mice [14]. The mice were deeply anesthetized using 80% carbon dioxide (CO_2_)/20% oxygen (O_2_) and had their dorsal hair shaved. An incision was then made to expose the left sixth lumbar spinal nerve by removing the L6 transverse process using a small scraper. The underlying fifth lumbar nerve root was then isolated and tightly ligated using 8-0 nylon thread, and the wound was sutured using muscle sutures (3-0 absorbable nylon) and skin sutures (3-0 nonabsorbable nylon). The surgical procedure in the sham group was identical, except that the fifth lumbar spinal nerve was not ligated or transected.

### 2.10. von Frey Test for Determining Mechanical Allodynia

Pain reactions were evaluated using the von Frey tests, which were carried out according to a previously described method for mice [14] using an electronic von Frey apparatus (IITC Inc., CA, USA). Briefly, we placed the mice individually in a transparent acrylic box (9 × 9 × 15 cm) with a wire mesh bottom. We allowed them to acclimate to their environment for at least 30 minutes. A mechanical stimulus was then applied from underneath to the plantar aspect of the hind limb, with a gradual increase in pressure. The end point was removal of the paw, followed by clear flinching movements. After paw withdrawal, the intensity of the pressure was automatically recorded. Each test was repeated three times at intervals of 5 minutes to gain an average value. The area under the time-effect curve (AUC) was calculated for each animal according to the following formula [14]: ([paw withdrawal pressure at each time point] − [paw withdrawal pressure at preoperative baseline value]) × time (days).

### 2.11. Quantification of IL-6 Levels

Blood samples were collected from the mice on day 13 following SNL surgery. Blood was mixed with 1.8 mg/mL EDTA and put on ice for less than 10 minutes. Plasma was collected by centrifugation at 1000x g for 10 minutes at 4°C and stored at −80°C until analysis. Cytokine levels were determined using a mouse IL-6 ELISA Kit (Abcam, ab100712).

### 2.12. Immunohistofluorescence of Spinal Astrocytes or Microglia

Spinal cord slices were taken from L5 spinal segments in mice. Sample preparation and observation were carried out according to a previous report [14]. To detect spinal astrocytes or microglia, sample sections were incubated with either mouse monoclonal anti-GFAP antibody (Millipore) or rabbit anti-iba1 antibody (Wako), as appropriate, diluted in PBS. Following primary antibody incubation, an appropriate secondary antibody conjugated to a fluorophore was used (goat anti-mouse-IgG FITC [Millipore, Cat #AP124F] or goat anti-rabbit IgG Alexa Fluor 488). Images of sample sections were captured using a Leica DMI 6000B inverted microscope with a Leica DFC 420 camera and MetaMorph software (Major Instruments Co., Ltd.). Fluorescence from GFAP and iba1 immunopositive cells were shown within the superficial dorsal horn.

### 2.13. Statistical Analysis

Statistical analysis was performed using one-way analysis of variance, and differences between treatments were assessed using Student's *t-*test. All *p*-values < 0.05 were regarded as statistically significant.

## 3. Results

### 3.1. The Presence and Cytotoxicity of E-A and E-S in HE-CE

**Figure 1:**
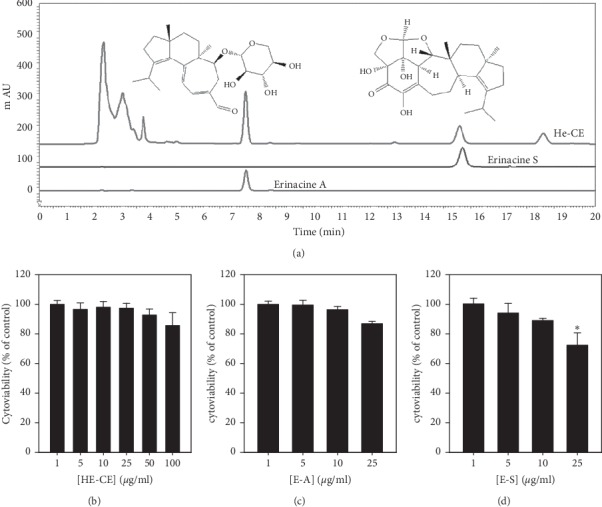
Composition of erinacine-A (E-A) and erinacine-S (E-S) within *Hericium erinaceus* mycelium crude extract (HE-CE) and their cytotoxicity in human SH-SY5Y cells. (a) High-performance liquid chromatography (HPLC) analysis of HE-CE content, shown with E-A and E-S standards. (b–d) Cytotoxicity of HE-CE (B), E-A (C), and E-S (D), as measured in human SH-SY5Y cells using the MTT assay. The cells were treated with HE compounds at various concentrations in a serum-free medium for 24 hours. ^*∗*^*p* < 0.05 between control/HE-CE (B), E-A (C), and E-S (D).

We performed HPLC analysis to investigate the composition of HE-CE.  Figure 1(a) shows the presence of E-A after about 7.4 minutes and that of E-S after about 15.1 minutes in HE-CE.  Figure 1(b) shows the cytotoxicity of HE-CE, E-A, and E-S. HE-CE, E-A, and E-S did not affect the viability of human SH-SY5Y cells at concentrations below 10 *μ*g/mL, while 25 *μ*g/mL E-S decreased cell viability.

### 3.2. HE-CE and E-S, but Not E-A, Decreased ATP-Induced Rise in [Ca^2+^]_c_

**Figure 2:**
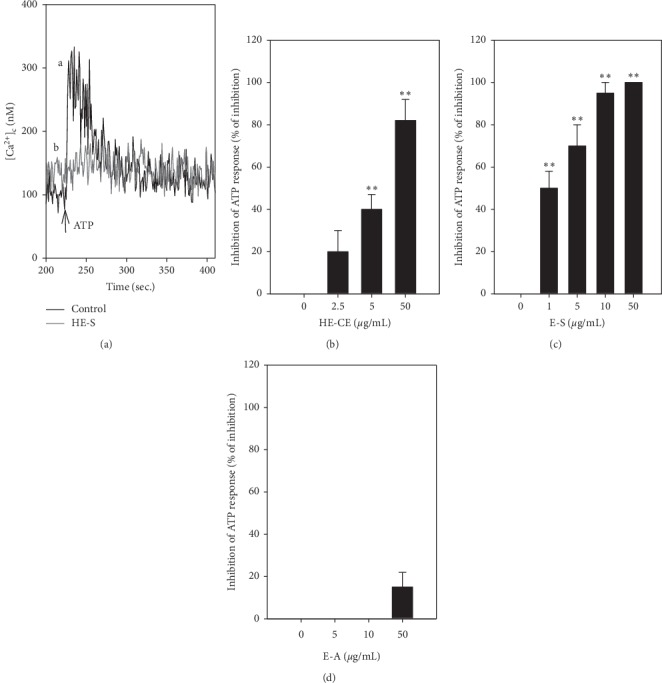
Effects of *Hericium erinaceus* (HE) on ATP-induced rise in [Ca^2+^]_c_ in human osteosarcoma HOS cells. (a) Cells treated with 0.1 mM ATP (↑) either without (curve a in black) or with (curve b in red) HE mycelium crude extract (HE-CE) (50 *μ*g/mL). (B-D) Effects of HE-CE (b), erinacine-S (E-S) (c), and erinacine-A (E-A) (d) on the ATP-induced rise in [Ca^2+^]_c_. Net change in ATP-induced rise in [Ca^2+^]_c_, calculated as the peak value minus the basal level. Data are presented as the inhibitory percentage, calculated as 100% (control) minus the % response in the presence of HE-CE, E-S, or E-A. All results were from three to five experiments, using different batches of cells, with duplicates for each experiment. ^*∗∗*^*p* < 0.01 between ATP/ATP + HE-CE, E-S, or E-A.

ATP is a well-known P2R agonist, and 0.1 mM ATP induced a transient rise in [Ca^2+^]_c_ with a peak of approximately 195 ± 32 nM, in human HOS cells (Figure 2(b)). HE-CE and E-S individually inhibited this ATP-induced rise in [Ca^2+^]_c_ in HOS cells in a dose-dependent manner, with the half maximal inhibitory concentration (IC_50_) values of 5 *μ*g/mL and 1 *μ*g/mL, respectively (Figure 2). E-A exerted a small inhibitory effect on the ATP-induced rise in [Ca^2+^]_c_. Specifically, at 50 *μ*g/mL, E-A showed an approximately 20% inhibitory effect. At 10 *μ*g/mL, E-S almost completely blocked the ATP-induced rise in [Ca^2+^]_c_ at 5 *μ*g/mL; E-S still exhibited potent blockage (approximately 75%). Thus, E-S was more effective than HE-CE, in this regard, and we propose that E-S is the active compound in HE-CE that inhibits the ATP-induced rise in [Ca^2+^]_c_ indicative of P2R-mediated signal transduction.

### 3.3. HE-CE Counteracted the Cellular Damage Induced by ATP

**Figure 3:**
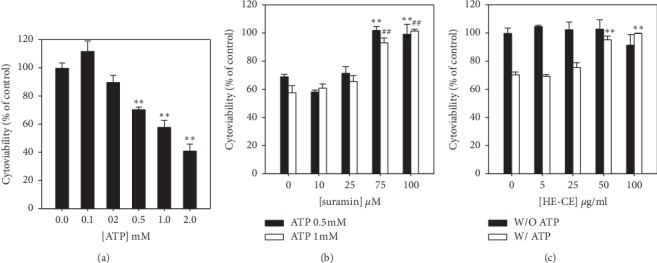
Effect of suramin and *Hericium erinaceus* mycelium crude extraction (HE-CE) on ATP-induced toxicity in human neuroblastoma SH-SY5Y cells. Cell viabilities were detected using the MTT assay. (a) Effect of ATP on cell viability. The cells were treated with ATP at various concentrations for 24 hours in a serum-free medium. ^*∗∗*^*p* < 0.01*vs.* vehicle control. (b) Effect of suramin on ATP-induced cytotoxicity. Cells were treated with 0.5 mM (black bars) and 1 mM ATP (open bars) in the presence of suramin at various concentrations for 24 hours. ^*∗∗*^*p* < 0.01 between 0.5 mM ATP/suramin+ 0.5 mM ATP; ^##^*p* < 0.01 between 1 mM ATP/suramin+ 1 mM ATP. (c) Effect of HE-CE on ATP-induced cytotoxicity. Cells were treated with HE-CE for 16 hours and then with 0.5 mM ATP (open bars) or no ATP (black bars) in the presence of HE-CE at various concentrations for 24 hours. ^*∗∗*^*p* < 0.01 between ATP/ATP + HE-CE.

At high concentrations, extracellular ATP causes neuronal damage [20]. We used human neuroblastoma SH-SY5Y cells to investigate the neuronal damage induced by ATP and the damage-preventing effects of ATP receptor (P2R) antagonists.  Figure 3(a) shows that ATP decreased the viability of human neuroblastoma SH-SY5Y cells in a dose-dependent manner. At 0.5 mM, ATP induced significant toxicity. Suramin, an antagonist of P2R, counteracted the toxicity induced by ATP at 0.5 and 1 mM. At 75 *μ*M, suramin completely blocked the ATP-induced toxicity (Figure 3(b)). HE-CE also counteracted ATP-induced cytotoxicity in a dose-dependent manner (Figure 3(c)). At 50 *μ*g/mL, HE-CE completely blocked both the ATP-induced toxicity (Figure 3) and the ATP-induced rise in [Ca^2+^]_c_ (Figure 2).

### 3.4. Body Weight of Mice following SNL Surgery

**Figure 4:**
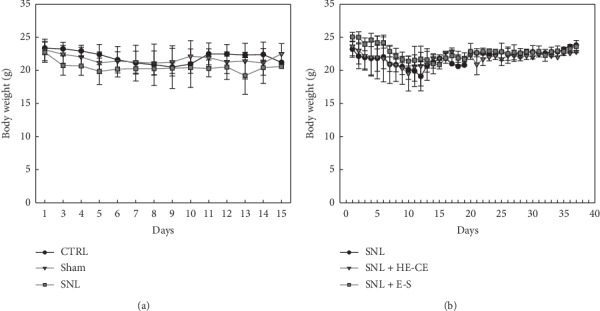
The body weight of mice after spinal nerve ligation (SNL) surgery or oral gavage with *Hericium erinaceus* (HE). (a) Body weight of control mice (black line with circles), sham-treated mice (red line with inverted triangles), and SNL-treated mice (green line with squares). (b) Body weight of SNL-treated mice. SNL-treated mice were fed with 5% glucose (black line with circles), HE mycelium crude extract (HE-CE) (red line with inverted triangles), and erinacine-S (E-S) (green line with squares). Surgery was carried out on day 1 following body weight measurements. Data are presented as mean ± standard deviation (5 mice/group).

All mice survived until the 18th day of the present study. There was no evidence of severe general toxicity until the end of this experiment. In general, body temperature remained unaltered and the animals' general health did not deteriorate when they were treated with SNL or fed with HE-CE or E-S. A small, nonsignificant reduction in body weight (−4.6% ± 8.0%; −1.1 ± 1.87 g/mouse) was seen immediately after SNL surgery (*p* > 0.05, ANOVA), and a reduction in body weight of -2.6% ± 6.5% and −0.2% ± 3.9% was found after feeding with E-S and HE-CE, respectively. There was no significant difference in body weight among control, sham, and SNL-treated groups (Figure 4(a), *p* > 0.05, two-way ANOVA).

### 3.5. SNL-Induced Neuropathic Pain

**Figure 5:**
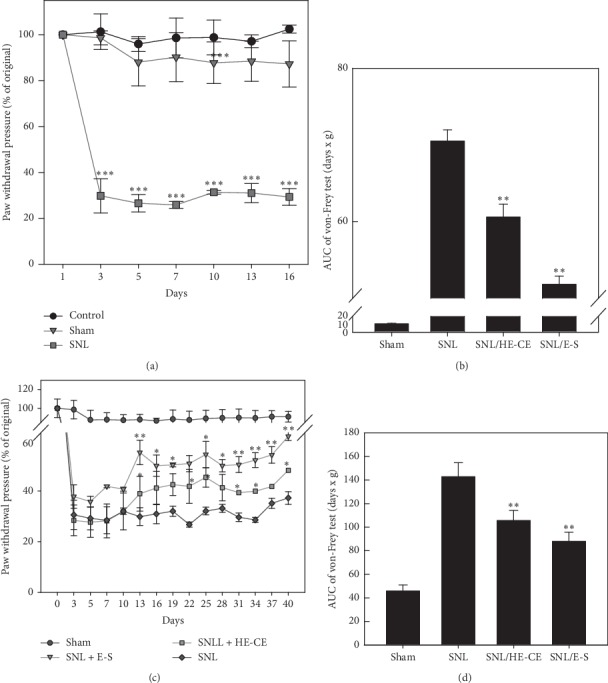
Effect of *Hericium erinaceus* (HE) extracts on spinal nerve ligation (SNL)-induced allodynia. Ipsilateral hind paw withdrawal pressure was determined every other day following SNL by the von Frey test. (a) Effect of SNL surgery and sham surgery on paw withdrawal pressure. (b) Effect of oral gavage with HE mycelium crude extract (HE-CE) (100 mg/kg/day) or erinacine-S (E-S) (30 mg/kg/day) on SNL-induced pressure. (C-D) Inverse area under the curve (AUC) values for corresponding curves from the von Frey tests during days 1–16 (c) and 17–40 (d). Data are presented as mean ± standard deviation (*n* = 5 ∗ 2). One-way analysis of variance was used to analyze the data. ^*∗∗∗*^*p* < 0.001*vs*. sham group in (A); ^*∗*^*p* < 0.05, ^*∗∗*^*p* < 0.01*vs*. SNL +5% glucose group in (B); and ^*∗*^*p* < 0.05, ^*∗∗*^*p* < 0.01*vs*. sham group in (C) and (D).

SNL-surgery induced mechanical allodynia, as measured using the von-Frey test. Withdrawal pressure for the ipsilateral hind paw decreased from 7.94 ± 0.79 g (*n* = 15) to 2.36 ± 0.55 g (*n* = 15) following SNL surgery (Figure 5), while the withdrawal pressure remained at 7.90 ± 0.69 g (*n* = 5) and 7.61 ± 0.79 g in the control and sham groups, respectively. This significant decrease in withdrawal pressure (*p* < 0.001) in the SNL-surgery group reflected neuropathic pain, which lasted until day 40. The sham treatment did not change the ipsilateral hind paw withdrawal pressure (Figure 5(a)), and the withdrawal pressure on the contralateral side in the SNL group remained similar to that of the control group until day 40 of this experiment (Figure 5(b)). These results indicate that L5-SNL induced the allodynia that prompted the ipsilateral hind paw withdrawal. SNL-induced mechanical allodynia served as a measure of neuropathic pain in the present study.

### 3.6. Effects of HE-CE and E-S on SNL-Induced Neuropathic Pain

The effects of HE on neuropathic pain were investigated by measuring the withdrawal pressure of the hind paw in the von Frey test following HE-CE (100 mg/kg/day) and E-S (30 mg/kg/day) feeding. Mice fed with either HE-CE or E-S showed a significant smaller decrease in the withdrawal pressure of the ipsilateral hind paw induced by SNL. On day 13, a significant increase in withdrawal pressure was measured in the mice given oral gavage of either HE-CE or E-S following SNL surgery (*p* < 0.05 and 0.01, resp.). These data support our hypothesis that HE possesses antiallodynic properties that can improve the chronic course of SNL-induced neuropathic pain. The corresponding AUCs of the time-effect curves were calculated and shown in Figures 5(c) and Figure 5(d). Throughout days 1–16, both HE-CE and E-S significantly reduced the increase in AUC induced by SNL (*p* < 0.001). The pain-relieving effects of E-S were significantly better than those of HE-CE (*p* < 0.01). Over days 17–40, E-S also significantly lowered the increase in AUC induced by SNL (*p* < 0.01), as did HE-CE (*p* < 0.01). Taken together, these data show that both HE-CE and E-S have an antiallodynic effect and that E-S was significantly more effective than HE-CE in this regard (*p* < 0.05).

### 3.7. HE-CE Reduced the SNL-Induced Elevation in IL-6 Levels

**Figure 6:**
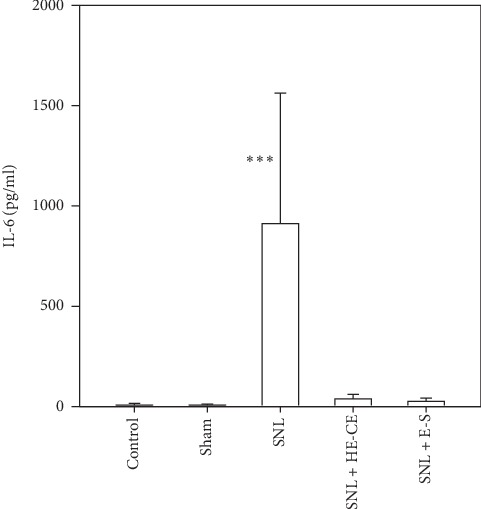
Effect of *Hericium erinaceus* mycelium crude extract (HE-CE) and erinacine-S (E-S) on spinal nerve ligation (SNL)-induced elevation of plasma IL-6 levels of mice. Plasma was collected on day 13 (*n* = 5–10). ^*∗∗∗*^*p* < 0.001 between SNL + HE-CE/E-S groups and SNL group.

IL-6 is upregulated following nerve injury, and spinal-nerve injury induces rapid production and release of IL-6 [29].  Figure 6 shows that SNL surgery, but not sham treatment, raised the levels of IL-6 in plasma significantly. Both HE-CE and E-S significantly suppressed the increase in IL-6 following L5-SNL.

### 3.8. HE-CE Suppressed the Activation of Astrocytes and Microglia after SNL Surgery

**Figure 7:**
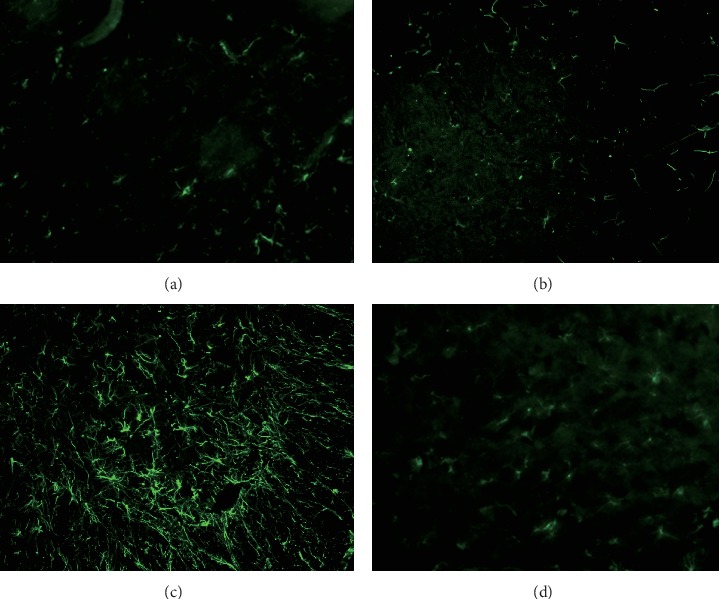
Effect of *Hericium erinaceus* mycelium crude extract (HE-CE) on spinal nerve ligation (SNL)-induced activation of astrocytes in the L5 spinal cord dorsal horn. Representative immunohistofluorescent images of astrocytes stained with glial fibrillary acidic protein (GFAP; a marker for activated astrocytes; green) on day 13 are shown for the following groups: control (a), SNL (ipsilateral) (b), sham (ipsilateral) (c), and SNL + HE-CE (ipsilateral) (d). Magnification: 200×.

**Figure 8:**
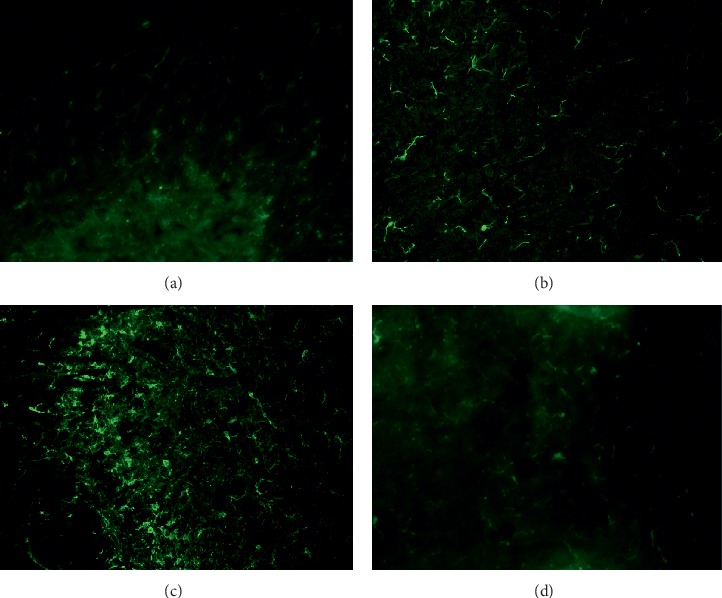
Effects of *Hericium erinaceus* mycelium crude extract (HE-CE) on spinal nerve ligation (SNL)-induced activation of microglia in the L5 spinal cord dorsal horn. Representative immunohistofluorescent images of microglia stained with ionized calcium-binding adaptor molecule-1 (iba1: a marker for activated microglia; green) on day 13 are shown for the following groups: Control (a), SNL (ipsilateral) (b), sham (ipsilateral) (c), and SNL + HE-CE (ipsilateral) (d). Magnification: 200×.

Activations of spinal astrocytes and microglia are involved during the initiation and maintenance of neuropathic pain. We carried out immunohistofluorescence studies to investigate changes in astrocytes and microglia in the dorsal spinal cord (L5) on the ipsilateral side on day 13 after SNL surgery. Tissue sections were stained using GFAP- and iba1-specific antibodies, which were then visualized using appropriate fluorescent-conjugated secondary antibodies to reveal the activation of astrocytes and microglia, respectively. Morphological changes in the astrocytes and microglia are shown in Figures 7 and 8, respectively.  Figure 7 shows that GFAP staining, in dicating GFAP-positive astrocytes, is greatly increased after SNL surgery (Figure 7(b)), but not in the control group (Figure 7(a)) or after sham surgery (Figure 7(c)). Daily oral gavage using HE-CE suppressed this SNL-induced activation of astrocytes to the level of control group (Figure 7(d)). Activation of microglia was assessed by iba1 staining on the ipsilateral side of the dorsal spinal cord on day 13 (Figure 8). The immunofluorescence for iba1 was markedly increased in the ipsilateral L5 spinal cord dorsal horn following SNL surgery (Figure 8(b)), but not in the controls (Figure 8(a)). There were some iba1-positive microglia in the sham-treated group (Figure 8(c)), but markedly fewer than in the SNL group. Daily oral gavage with HE-CE suppressed the SNL-induced activation of microglia (Figure 8(d)) to the level seen in controls.

### 3.9. Both P2X4 and P2X7 Increased following SNL Surgery

**Figure 9:**
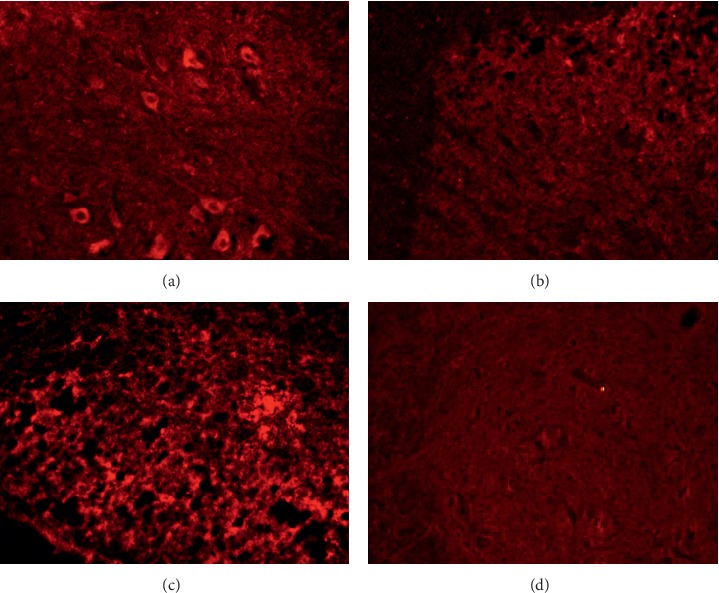
Immunofluorescence for P2X4 expression on the ipsilateral (a) and contralateral (b) sides in the spinal cord dorsal horn, and P2X7 expression in the ipsilateral (c) and contralateral (d) sides in the spinal nerve ligation (SNL) group. Magnification: 100×. (a) P2X4 (SNL-ipsilateral). (b) P2X4 (SNL-contralateral). (c) P2X7 (SNL-ipsilateral). (d) P2X7 (SNL-contralateral).

The expression levels of P2X4 and P2X7 in the spinal cord dorsal horn were observed using immunofluorescence measurements after SNL-surgery.  Figure 9 shows that the levels of P2X4 and P2X7 were higher on the SNL-ipsilateral side than on the contralateral side on day 13.

## 4. Discussion

The present study demonstrated that HE-CE can reduce both L5-SNL-induced mechanical allodynia in mice and ATP-induced rises in [Ca^2+^]_c_ in a human cell line. The rise in [Ca^2+^]_c_ induced by ATP—an agonist of P2R—may reflect the cellular activity of P2Rs in pain transduction. SNL surgery causes neuropathic pain by inflicting neuronal damage on the dorsal root ganglion, which increases mechanical allodynia. The von Frey tests in the present study showed that HE-CE significantly suppresses SNL-induced allodynia, thus characterizing HE-CE as an analgesic. HE-CE also reduced the increase in IL-6 levels and the activation of astrocytes and microglia following SNL surgery, which illustrates that HE-CE can suppress neuroinflammation.

We previously reported that HE-CE inhibited P2R-coupled Ca^2+^ signaling and heat-induced pain [21]. The current study showed that HE-CE and E-S can completely inhibit the ATP-induced rise in [Ca^2+^]_c_ in HOS cells expressing the P2R subtypes P2X_1,4,5,6,7_R and P2Y_2,4,5,9,11_R. Our data, thus, suggest that both HE-CE and E-S inhibit P2Rs, which transduce pain from the peripheral to the central nervous system [7]. The levels of P2X4 and P2X7 in the spinal cord play important roles in the generation and maintenance of neuropathic pain [7,10,13,30]. Pharmacological blockade of spinal P2X4 receptors can relieve tactile allodynia [10]. Relatedly, *α*,*β*-methylene, a P2X4R agonist, rapidly initiates nociceptive behavior [5]. Blockage of P2X4R gene expression using an antisense oligonucleotide attenuated morphine-induced tolerance and hyperalgesia [31], while disruption of P2X7 purinoceptor gene expression blocked chronic inflammatory and neuropathic pain in mice [32]. These findings highlight the roles of P2X4R of P2X7R in neuropathic pain. In the present study, and in our previous report [21], we provided data demonstrating that HE completely suppressed the activities of P2X_1,4,5,6,7_R and P2Y_2,4,5,9,11_R in a human cell line. We propose that the suppression of P2R function can block P2R pain transmission, conferring analgesic properties.

The present study used L5-SNL surgery, which is a highly reproducible procedure that results in little surrounding tissue damage [15]. The neuropathic symptoms generated by the SNL model mimic those of human patients suffering from causalgia following a nerve injury [12]. A previous study reported that SNL mice retained neuropathic pain symptoms for 2 months [14,33]. The present study found that the SNL surgery in mice induced a high and stable level of von-Frey-measured pain behavior that persisted for 37 days. SNL-induced neuropathic pain is related to P2R activity [8]. In one study, the expression and production of spinal P2X7R increased following SNL surgery [34], while in another, the expression levels of P2X7R in the spinal cord segment were higher in the nerve surgery group than in the sham group [35,36]. Finally, P2X4 mRNA expression levels in the spinal horn increased following SNL surgery in mice [37]. The present study also found that the SNL surgery site expressed P2X4 and P2X7 at higher levels than the contralateral side (Figure 9). Because P2X4 and P2X7 were upregulated after SNL surgery, causing chronic pain, blockage of P2XR by HE-CE and E-S might play a role in relieving SNL-induced neuropathic pain.

We found a massive elevation in plasma IL-6 levels following SNL surgery in mice. Other studies support this finding. Increases in IL-6 levels are coupled with nerve injury [38]. SNL surgery induced both spinal damage and a corresponding plasma IL-6 levels [14]. One study found that elevation in IL-6 levels is responsible for nerve injury-induced mechanical hypersensitivity and pain maintenance in rodents [38,39]. In the present study, both HE-CE and E-S suppressed the SNL-induced massive elevation in IL-6 levels (Figure 6), illustrating the anti-inflammatory action of both substances. Some reports support our finding that HE has immunosuppressive characteristics. For example, HE can block elevated IL-6 induced by stress in mice [40]. It can also reduce lipopolysaccharide-induced IL-6 increases in both RAW264 macrophages [41] and an animal stroke model [42]. In this regard, Li proposed a possible link between relief from neuropathic pain and suppression of IL-6 levels [4] and that the suppressive effects of HE-CE and E-S on SNL-induced IL-6 elevation may explain their analgesic properties. P2X7 activation regulates the processing and release of cytokines [36], and increased P2X4R expression is coupled with neuronal inflammation in the ipsilateral spinal cord following nerve injury [37]. Since P2R blockage can suppress inflammation, we propose that HE suppresses the SNL-induced elevation in IL-6 levels that might occur by blocking P2R function through HE-CE.

In the present study, SNL surgery activated astrocytes and microglia at the site of surgery. HE-CE counteracted the elevated levels of GFAP in astrocytes and of iba1 in microglia following SNL surgery, illustrating that HE has neuroinflammation-suppressing properties. SNL surgery induces neuroinflammation and neuropathic pain by activating astrocytes and microglia, as supported by other studies. The activation of astrocytes and microglia is associated with the initiation and maintenance of neuropathic pain [14,17,43]. P2X4 and P2X7 were present in astrocytes and microglia in the present study; these receptors are known to transmit neuropathic pain [30,36]. In a previous study, chronic inflammation and neuropathic pain could not be induced and maintained when the P2X7 purinoceptor gene was disrupted [32]. The action of HE-CE as a P2R antagonist may mediate its anti-inflammatory and neuropathic pain reliever properties. P2R is a well-defined therapeutic target for inflammation and neuropathic pain [3]. The present study found that the SNL surgery side showed more P2X4 and P2X7, increased IL-6 levels, and activation of astrocytes and microglia, all of which play a role in SNL-induced neuropathic pain. HE-CE suppressed all these SNL-induced phenomena and relieved neuropathic pain. As such, HE-CE is potent to alleviate neuropathy.

## 5. Conclusions

Our data strongly indicate that HE has analgesic properties, because HE-CE suppressed L5-SNL-induced neuropathic pain and elevated plasma IL-6. Astrocyte and microglial activation may initiate and maintain neuropathic pain and neuroinflammation. HE-CE suppressed both IL-6 elevation and the activation of astrocytes and microglia following L5-SNL, and it blocked P2X4 and P2X7 activity in a human cell line. P2X4 and P2X7 are present in astrocytes and microglia, and they induce neuropathic pain and inflammation. We propose that HE-CE acts as an analgesic to relieve neuropathic pain through its properties as a P2R antagonist and anti-inflammatory.

## Data Availability

All data generated or analyzed during this study are included in this published article and its supplementary information files.

## References

[1] Burnstock G. (2006). Historical review: ATP as a neurotransmitter. *Trends in Pharmacological Sciences*.

[2] Cook S. P., McCleskey E. W. (2002). Cell damage excites nociceptors through release of cytosolic ATP. *Pain*.

[3] Munoz F. M., Gao R., Tian Y. (2017). Neuronal P2X7 receptor-induced reactive oxygen species production contributes to nociceptive behavior in mice. *Scientific Reports*.

[4] Li X., Kang L., Li G. (2013). Intrathecal leptin inhibits expression of the P2X2/3 receptors and alleviates neuropathic pain induced by chronic constriction sciatic nerve injury. *Molecular Pain*.

[5] Stanfa L. C., Kontinen V. K., Dickenson A. H. (2000). Effects of spinally administered P2X receptor agonists and antagonists on the responses of dorsal horn neurones recorded in normal, carrageenan-inflamed and neuropathic rats. *British Journal of Pharmacology*.

[6] Trang T., Beggs S., Salter M. W. (2006). Purinoceptors in microglia and neuropathic pain. *Pflügers Archiv—European Journal of Physiology*.

[7] Burnstock G. (2016). Purinergic mechanisms and pain. *Pharmacological Mechanisms and the Modulation of Pain*.

[8] Kim C., Chung J. M., Chung K. (2003). Changes in the gene expression of six subtypes of P2X receptors in rat dorsal root ganglion after spinal nerve ligation. *Neuroscience Letters*.

[9] Colloca L., Ludman T., Bouhassira D. (2017). Neuropathic pain. *Nature Reviews. Disease Primers*.

[10] Tsuda M., Masuda T., Tozaki-Saitoh H., Inoue K. (2013). P2X4 receptors and neuropathic pain. *Frontiers in Cellular Neuroscience*.

[11] Kim S. H., Chung J. M. (1992). An experimental model for peripheral neuropathy produced by segmental spinal nerve ligation in the rat. *Pain*.

[12] Kumar A., Kaur H., Singh A. (2018). Neuropathic Pain models caused by damage to central or peripheral nervous system. *Pharmacological Reports*.

[13] M’Dahoma S., Bourgoin S., Kayser V. (2014). Spinal cord transection-induced allodynia in rats--behavioral, physiopathological and pharmacological characterization. *BMC Complementary and Alternative Medicine*.

[14] Yang P. P., Yeh G. C., Huang E. Y. (2015). Effects of dextromethorphan and oxycodone on treatment of neuropathic pain in mice. *Journal of Biomedical Science*.

[15] LaBuda C. J., Little P. J. (2005). Pharmacological evaluation of the selective spinal nerve ligation model of neuropathic pain in the rat. *Journal of Neuroscience Methods*.

[16] Jin S.-X., Zhuang Z.-Y., Woolf C. J., Ji R.-R. (2003). p38 mitogen-activated protein kinase is activated after a spinal nerve ligation in spinal cord microglia and dorsal root ganglion neurons and contributes to the generation of neuropathic pain. *The Journal of Neuroscience*.

[17] Zhuang Z.-Y., Wen Y. R., Zhang D. R. (2006). A peptide c-Jun N-terminal kinase (JNK) inhibitor blocks mechanical allodynia after spinal nerve ligation: respective roles of JNK activation in primary sensory neurons and spinal astrocytes for neuropathic pain development and maintenance. *Journal of Neuroscience*.

[18] Zou L., Gong Y., Liu S., Liang S. (2019). Natural compounds acting at P2 receptors alleviate peripheral neuropathy. *Brain Research Bulletin*.

[19] Volonte C., Amadio S., Cavaliere F., D’Ambrosi N., Vacca F., Bernardi G. (2003). Extracellular ATP and neurodegeneration. *Current Drug Target -CNS & Neurological Disorders*.

[20] Chao C.-C., Chan P., Kuo C.-S. (2014). Protection of differentiated neuronal NG108-15 cells from P2X7 receptor-mediated toxicity by taurine. *Pharmacological Reports*.

[21] Liu P.-S., Chueh S.-H., Chen C.-C., Lee L.-Y., Shiu L.-Y. (2017). Lion’s mane medicinal mushroom, *Hericium erinaceus* (agaricomycetes), modulates purinoceptor-coupled calcium signaling and murine nociceptive behavior. *International Journal of Medicinal Mushrooms*.

[22] Hu J.-H., Li I.-C., Lin T.-W. (2019). Absolute bioavailability, tissue distribution, and excretion of erinacine S in *Hericium erinaceus* mycelia. *Molecules*.

[23] Tzeng T.-T., Chen C.-C., Chen C.-C. (2018). The cyanthin diterpenoid and sesterterpene constituents of *Hericium erinaceus* mycelium ameliorate alzheimer’s disease-related pathologies in APP/PS1 transgenic mice. *International Journal of Molecular Sciences*.

[24] Li I.-C., Chen Y.-L., Lee L.-Y. (2014). Evaluation of the toxicological safety of erinacine A-enriched *Hericium erinaceus* in a 28-day oral feeding study in Sprague-Dawley rats. *Food and Chemical Toxicology*.

[25] Chen C.-C., Tzeng T.-T., Chen C.-C. (2016). Erinacine S, a rare sesterterpene from the mycelia of *Hericium erinaceus*. *Journal of Natural Products*.

[26] Yan R. M., Chiung Y. M., Pan C. Y., Liu J. H., Liu P. S. (2008). Effects of dichlorobenzene on acetylcholine receptors in human neuroblastoma SH-SY5Y cells. *Toxicology*.

[27] Liu P.-S., Chen C.-Y. (2010). Butyl benzyl phthalate suppresses the ATP-induced cell proliferation in human osteosarcoma HOS cells. *Toxicology and Applied Pharmacology*.

[28] McGrath J., Drummond G., McLachlan E., Kilkenny C., Wainwright C. (2010). Guidelines for reporting experiments involving animals: the ARRIVE guidelines. *British Journal of Pharmacology*.

[29] Thacker M. A., Clark A. K., Marchand F., McMahon S. B. (2007). Pathophysiology of peripheral neuropathic pain: immune cells and molecules. *Anesthesia & Analgesia*.

[30] Beggs S., Trang T., Salter M. W. (2012). P2X4R+ microglia drive neuropathic pain. *Nature Neuroscience*.

[31] Horvath R. J., Romero-Sandoval A. E., De Leo J. A. (2010). Inhibition of microglial P2X4 receptors attenuates morphine tolerance, Iba1, GFAP and *μ* opioid receptor protein expression while enhancing perivascular microglial ED2. *Pain*.

[32] Chessell I. P., Hatcher J. P., Bountra C. (2005). Disruption of the P2X7 purinoceptor gene abolishes chronic inflammatory and neuropathic pain. *Pain*.

[33] Kiso T., Watabiki T., Tsukamoto M. (2008). Pharmacological characterization and gene expression profiling of an L5/L6 spinal nerve ligation model for neuropathic pain in mice. *Neuroscience*.

[34] Yang J., Park K. S., Yoon J. J. (2016). Anti-allodynic effect of intrathecal processed Aconitum jaluense is associated with the inhibition of microglial activation and P2X7 receptor expression in spinal cord. *BMC Complementary Medicine and Therapies*.

[35] Zhang W., Liu Y., Sun Y., Liu Z. (2019). Effects of microencapsulated olfactory ensheathing cell transplantation on neuropathic pain and P2X7 receptor expression in the L4-5 spinal cord segment. *Neuroscience Letters*.

[36] Lu W., Albalawi F., Beckel J. M., Lim J. C., Laties A. M., Mitchell C. H. (2017). The P2X7 receptor links mechanical strain to cytokine IL-6 up-regulation and release in neurons and astrocytes. *Journal of Neurochemistry*.

[37] Aby F., Whitestone S., Landry M., Ulmann L., Fossat P. (2018). Inflammatory-induced spinal dorsal horn neurons hyperexcitability is mediated by P2X4 receptors. *Pain Reports*.

[38] Arruda J. L., Colburn R. W., Rickman A. J., Rutkowski M. D., DeLeo J. A. (1998). Increase of interleukin-6 mRNA in the spinal cord following peripheral nerve injury in the rat: potential role of IL-6 in neuropathic pain. *Molecular Brain Research*.

[39] Quarta S., Vogl C., Constantin C. E. (2011). Genetic evidence for an essential role of neuronally expressed IL-6 signal transducer gp130 in the induction and maintenance of experimentally induced mechanical hypersensitivity in vivo and in vitro. *Molecular Pain*.

[40] Chiu C.-H., Chyau C.-C., Chen C.-C. (2018). Erinacine A-enriched Hericium erinaceus mycelium produces antidepressant-like effects through modulating BDNF/PI3K/Akt/GSK-3*β* signaling in mice. *International Journal of Molecular Sciences*.

[41] Mori K., Ouchi K., Hirasawa N. (2015). The anti-inflammatory effects of lion’s mane culinary-medicinal mushroom, *Hericium erinaceus* (higher basidiomycetes) in a coculture system of 3T3-L1 adipocytes and RAW264 macrophages. *International Journal of Medicinal Mushrooms*.

[42] Choi H.-N., Kang M.-J., Lee S.-J., Kim J.-I. (2014). Ameliorative effect of myricetin on insulin resistance in mice fed a high-fat, high-sucrose diet. *Nutrition Research and Practice*.

[43] Tsuda M. (2016). Microglia in the spinal cord and neuropathic pain. *Journal of Diabetes Investigation*.

